# Atypical Spontaneous Pneumothorax in an Older Obese Female: A Case Report

**DOI:** 10.7759/cureus.110053

**Published:** 2026-06-01

**Authors:** Farheen A Rasheed, Michael D Brackenrich

**Affiliations:** 1 Medicine, Edward Via College of Osteopathic Medicine, Blacksburg, USA; 2 Family Medicine, Brackenrich Family Practice, Rich Creek, USA

**Keywords:** apical pulmonary bleb, chest tube thoracostomy, primary spontaneous pneumothorax, secondary spontaneous pneumothorax, smoking history, spontaneous pneumothorax, tobacco use

## Abstract

Spontaneous pneumothorax results from the accumulation of air in the pleural space, leading to partial or complete collapse of the affected lung. Primary spontaneous pneumothorax (PSP) classically occurs in young, tall, thin males without clinically apparent underlying lung disease, whereas secondary spontaneous pneumothorax (SSP) occurs in association with structural pulmonary pathology. Tobacco use is a well-established risk factor for spontaneous pneumothorax and may contribute to structural changes that persist after smoking cessation. We present an atypical case of spontaneous pneumothorax in a 65-year-old obese female with a remote smoking history who presented with acute dyspnea and right-sided pleuritic chest pain. Imaging demonstrated a large right-sided pneumothorax and a 1.7 cm apical bleb. The patient underwent chest tube thoracostomy with successful lung re-expansion and clinical improvement. Although the patient carried a documented clinical diagnosis of PSP, occult SSP could not be fully excluded given the patient’s prior tobacco exposure and structural pulmonary abnormality. This case highlights the diagnostic overlap that may exist between PSP and SSP in patients who do not fit the classic demographic profile.

## Introduction

Pneumothorax occurs when air accumulates in the pleural cavity, resulting in partial or complete lung collapse [1,2]. Spontaneous pneumothorax is traditionally classified as primary spontaneous pneumothorax (PSP), which occurs in the absence of clinically apparent lung disease, or secondary spontaneous pneumothorax (SSP), which occurs in association with underlying pulmonary pathology [3-6]. PSP commonly presents in young, tall, thin male patients between 20 and 30 years of age and is strongly associated with tobacco exposure [5,7,8].

The pathophysiology of PSP is thought to involve rupture of subpleural blebs or bullae [7,9]. Blebs are small air pockets that form on the surface of the lung, and bullae are air-filled cavities within the lung [10]. When blebs rupture, they allow inhaled air to travel from the airways to the thoracic cavity, disrupting the negative intrapleural pressure, which can result in pneumothorax [10]. The etiology of blebs and bullae is unknown [7]. Smoking has been proposed to contribute to airway inflammation, degradation of pulmonary elastic fibers through neutrophil- and macrophage-mediated inflammation, and pathological changes in the small airways that may promote bleb formation and air leakage into the lung interstitium [6,8,11]. However, blebs and bullae may also occur in nonsmokers, and the distinction between PSP and smoking-related SSP may not always be clearly defined in patients with prior tobacco exposure and structural pulmonary abnormalities [8,9]. Additionally, not all patients present with blebs or bullae, and an alternative explanation is leakage of air from pores in the pleura [9].

Limited literature exists describing spontaneous pneumothorax presentations in older obese former smokers without previously diagnosed chronic lung disease. This report describes an atypical presentation of spontaneous pneumothorax in an older obese female with a remote smoking history and CT-demonstrated apical bleb formation. Although the patient carried a documented diagnosis of PSP, this case highlights the diagnostic overlap that may exist between PSP and SSP in former smokers with structural pulmonary abnormalities. The patient was successfully managed with chest tube thoracostomy, in accordance with the most current British Thoracic Society Guidelines for Pleural Disease [12]. Conservative management, which is the preferred treatment, was unsuitable due to symptomatic hypoxia and clinical presentation. This case contributes to the existing literature by emphasizing the importance of considering spontaneous pneumothorax in patients who do not fit the classic PSP demographic profile while also highlighting the ongoing uncertainty regarding the long-term pulmonary effects of prior tobacco exposure.

## Case presentation

The patient in this case is a 65-year-old Caucasian female with a past medical history of morbid obesity (BMI: 40.6 kg/m²), mixed hyperlipidemia, hypertension, gastroesophageal reflux disease, fibromyalgia, anxiety, and insomnia. Current medications included semaglutide, losartan potassium 50 mg oral tablet daily, omeprazole 40 mg oral capsule before breakfast, bupropion hydrochloride 300 mg oral tablet daily, and zolpidem tartrate 12.5 mg extended-release oral tablet, one at bedtime as needed. The patient reported a 20-pack-year smoking history from early adulthood until smoking cessation approximately 20 years prior to presentation. She had no previously diagnosed chronic pulmonary disease.

The patient presented to her primary care provider (PCP) on November 6, 2025, with concerns of progressively worsening shortness of breath over two days. She also reported right-sided chest and rib pain in an area where she previously experienced panniculitis. She had self-administered leftover steroids from a prior panniculitis flare without symptomatic improvement but could not recall the medication name or dosage. She denied fever, chills, cough, or chest trauma.

Vital signs at presentation included a temperature of 97.8°F, a blood pressure of 139/89 mmHg, a pulse of 100 beats per minute, and an oxygen saturation of 86% on room air. Oxygen supplementation with 2 L/min via nasal cannula improved oxygen saturation to 90%. The patient appeared in mild to moderate respiratory distress compared to baseline but was able to speak comfortably while seated on supplemental oxygen. Physical examination demonstrated absent breath sounds throughout the right lung field, with preserved breath sounds on the left. The remainder of the physical examination was unremarkable. Due to concern for possible pneumothorax or large pulmonary embolism, the patient was referred immediately to the ED.

**Figure 1:**
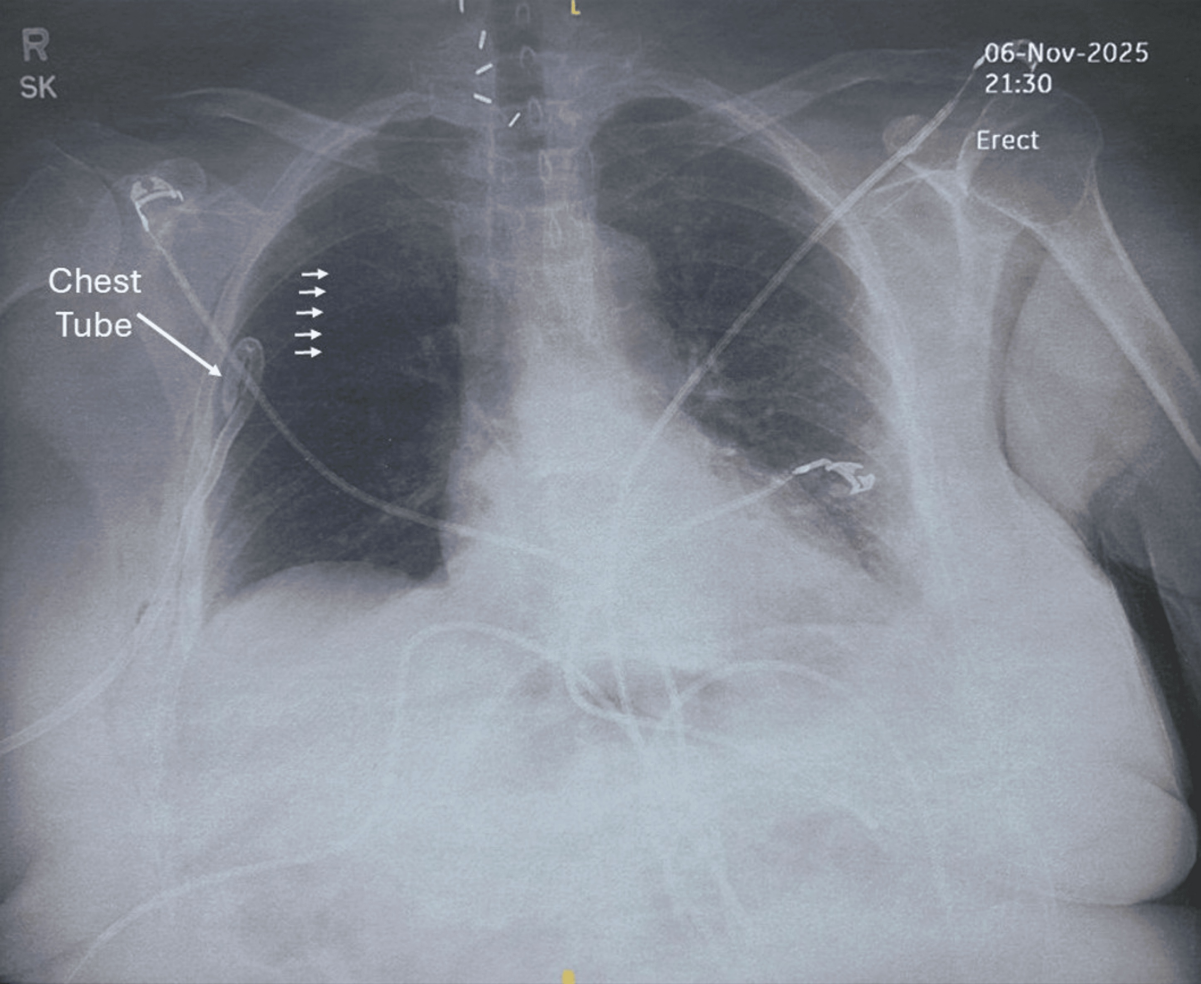
Chest radiograph demonstrating a large right-sided pneumothorax (small arrows) with pigtail chest tube placement

**Figure 2:**
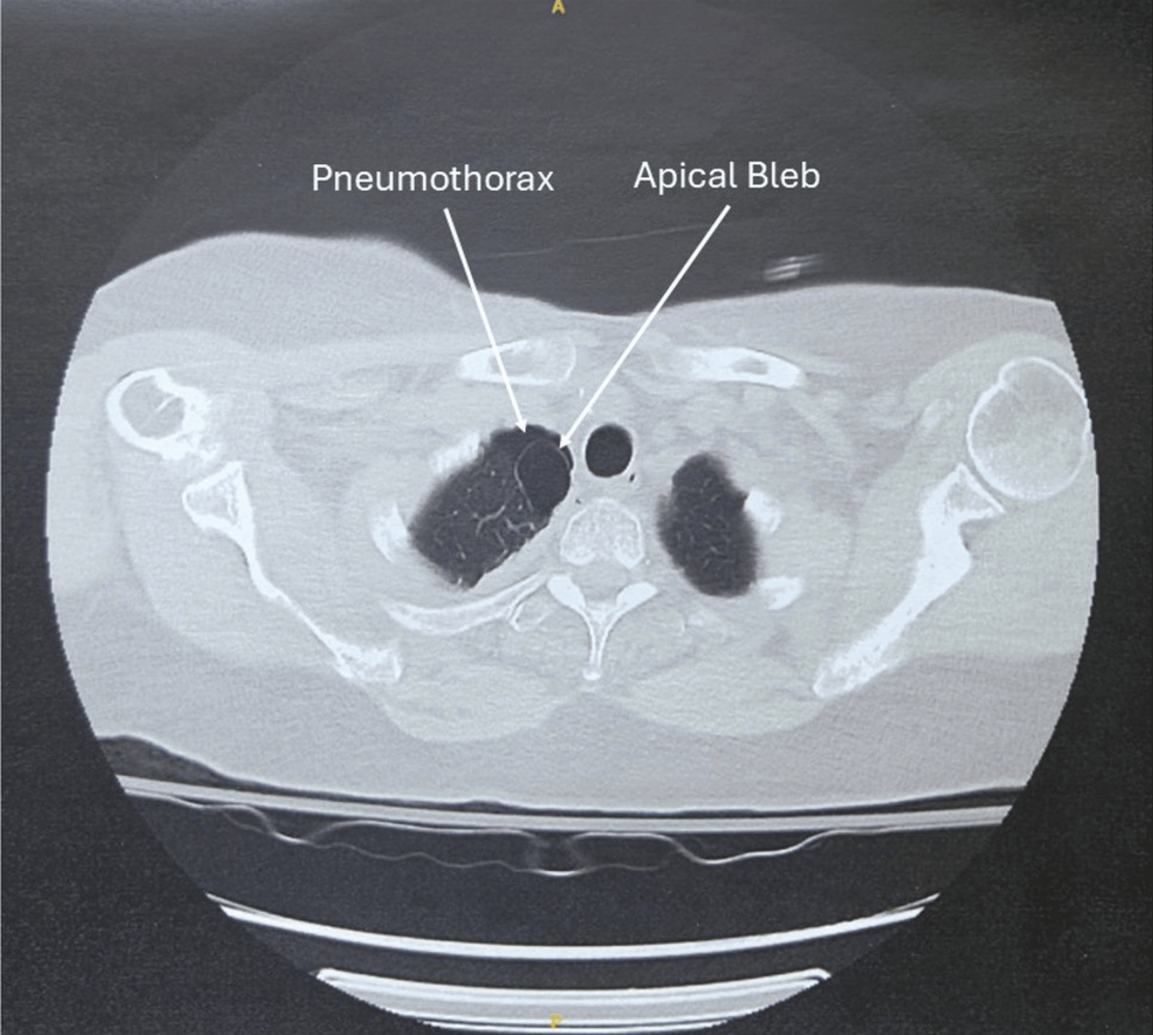
CT chest, axial view, demonstrating a residual small right apical pneumothorax and adjacent 1.7 cm apical bleb

Chest radiography at the ED demonstrated a large right-sided pneumothorax without tension physiology. Chest tube thoracostomy using a pigtail drainage catheter was performed for pleural decompression (Figure 1). A follow-up chest CT demonstrated good lung re-expansion and only a small right apical pneumothorax, significantly decreased in size, with a right-sided pleural tube in place. Furthermore, the scan showed a 1.7 cm right apical bleb (Figure 2); this finding suggests the possibility of a ruptured apical bleb as the etiology of the pneumothorax. No definitive diffuse emphysematous or bullous lung disease was identified on available imaging.

The patient was subsequently transferred from the community hospital to a tertiary care facility. Upon arrival at the second facility, a repeat chest CT confirmed appropriate positioning of the pleural pigtail catheter, with a residual tiny right apical pneumothorax involving less than 5% lung volume, trace right pleural effusions, and bibasilar atelectatic changes. A CBC with automated differential indicated an elevated WBC count of 14,730 cells/µL and an elevated neutrophil percentage of 76.9% (Table 1). Although no definitive pulmonary consolidation was identified on imaging, empiric antibiotic therapy was initiated because of concerns for a possible early infectious pulmonary process in the setting of leukocytosis, neutrophilia, and respiratory symptoms. Initial therapy with azithromycin was transitioned to doxycycline based on prior patient tolerance and outpatient treatment history.

**Table 1:** Selected clinically relevant laboratory findings

Test component	Patient value	Unit	Reference value
WBC	14.73 (high)	× 10³/µL	4.5-10.5
Platelet count	519 (high)	× 10³/µL	130-385
Neutrophils	76.9 (high)	Percent	40.0-75.0
Absolute neutrophil count	11.33 (high)	× 10³/µL	1.5-8.0

**Figure 3:**
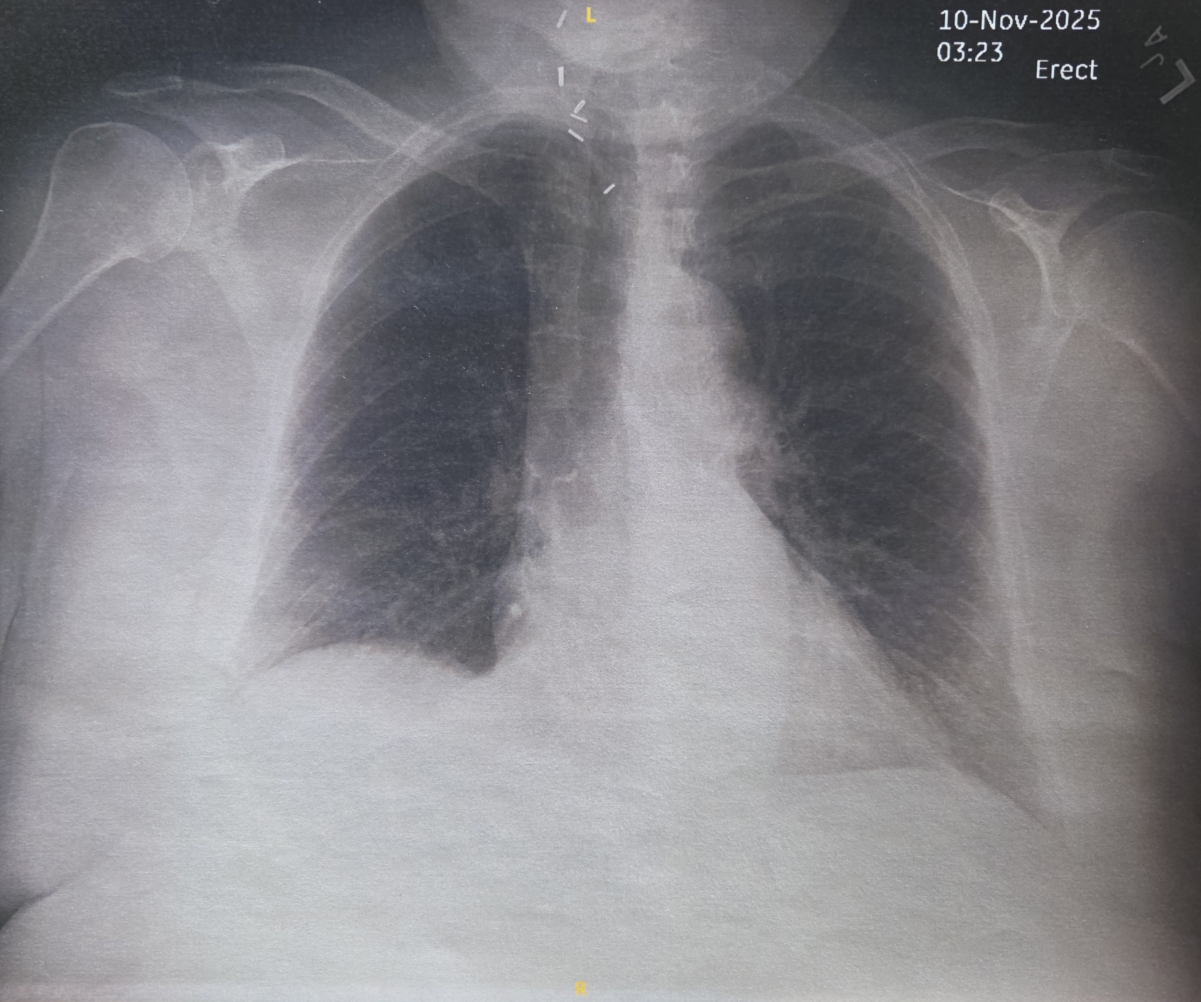
Chest radiograph demonstrating resolved right-sided pneumothorax

Additional inpatient treatment included peripheral intravenous fentanyl citrate 50 µg and morphine 4 mg for pain management, as well as DuoNeb as needed. Pulmonary medicine, as well as physical therapy, occupational therapy, and case management, were consulted on November 7, 2025. The chest tube was inadvertently displaced during transfer on November 9, 2025. Petroleum gauze dressing was applied to the insertion site, and a repeat chest radiograph demonstrated complete resolution of the pneumothorax without recurrence of symptoms (Figure 3). The patient remained on supplemental oxygen during hospitalization and was successfully weaned off oxygen prior to discharge after passing a walk test. A repeat CBC with differential indicated a WBC count within normal limits following completion of the antibiotic course.

The patient was discharged home in a hemodynamically stable condition on November 10, 2025, with 12 tablets of Lortab 5/325 mg prescribed for severe pain. She was instructed to follow up with her PCP for ongoing management. Review of the available medical records did not identify pulmonary function testing, diffusion studies, alpha-1 antitrypsin evaluation, thoracic surgical consultation, or planned pulmonary follow-up. These absent studies limited the ability to fully exclude subtle underlying emphysematous or bullous lung disease.

At outpatient follow-up, the patient demonstrated no evidence of recurrent pneumothorax or respiratory compromise. Physical examination revealed clear bilateral breath sounds without dyspnea. The patient expressed anxiety regarding recurrence and reduced physical activity following hospitalization. She was counseled regarding recurrence risk, symptom recognition, smoking avoidance, breathing exercises, and the importance of seeking urgent medical evaluation should symptoms recur.

## Discussion

Tobacco smoke exposure increases the risk of PSP in proportion to smoking duration and quantity, likely through the formation of blebs and bullae; however, blebs and bullae can also occur in nonsmokers [8]. In a study by Cheng et al., 80% of both smokers and nonsmokers experiencing a first episode of PSP had blebs or bullae on high-resolution CT imaging [8]. There was no statistical difference in pathological abnormalities between the two groups, except for respiratory bronchiolitis and tobacco-related pigmentation in smokers, which may contribute to increased recurrence rates [8]. These findings suggest that while structural abnormalities are common in PSP regardless of smoking status, tobacco exposure may produce additional inflammatory and small airway changes that influence disease progression. That being said, smoking cessation substantially lowers the risk of recurrence [13].

Earlier studies have also highlighted the lasting impact of tobacco exposure on PSP risk. Bense et al. examined the relationship between smoking habits and the occurrence of spontaneous pneumothorax [14]. The researchers found that smoking increased the relative risk of a first spontaneous pneumothorax by approximately ninefold in women and 22-fold in men [14]. Additionally, Kim et al. conducted a case-crossover study, which found that ex-smokers had increased odds of pneumothorax compared to nonsmokers, suggesting that prior tobacco exposure may confer persistent susceptibility, even after cessation [15]. Collectively, these findings indicate that the effects of smoking on small airway structures may not fully reverse after cessation. Despite this, there remains limited data specifically examining the prevalence and outcomes of spontaneous pneumothorax in former smokers.

This case highlights an atypical presentation of spontaneous pneumothorax in an older obese female with a remote smoking history, and CT demonstrated apical bleb formation. Importantly, the presence of an apical bleb alone does not definitively establish smoking-related SSP, as blebs may occur in both smokers and nonsmokers [8]. However, the patient’s smoking history and structural pulmonary abnormality raise the possibility of underlying smoking-related pulmonary changes that had not previously been clinically recognized.

Although the patient carried a documented diagnosis of PSP, this case illustrates the diagnostic overlap that may exist between PSP and SSP in former smokers. The patient had no previously diagnosed chronic pulmonary disease; however, pulmonary function testing, diffusion studies, high-resolution CT characterization beyond the index imaging, and alpha-1 antitrypsin evaluation were not performed. Additionally, while chest imaging did not demonstrate definitive pulmonary consolidation, infection-associated pneumothorax cannot be completely excluded. Empiric antibiotic therapy was initiated because of leukocytosis, neutrophilia, bibasilar atelectatic changes, and clinical concern for a possible early infectious pulmonary process. However, the available findings were nonspecific, and infection was not conclusively established during the hospitalization. Therefore, this case may exist along a clinical spectrum between PSP and SSP rather than fitting neatly into a single classification category.

The patient’s management was generally consistent with contemporary guideline-supported treatment for symptomatic spontaneous pneumothorax. While the current British Thoracic Society Guidelines support conservative management in minimally symptomatic patients, chest tube thoracostomy remains appropriate in patients with symptomatic hypoxia or those unsuitable for conservative management strategies [12]. The patient demonstrated successful clinical and radiographic improvement following chest tube placement.

This report has several limitations inherent to case report methodology. Causality between remote smoking exposure and pneumothorax development cannot be established from a single observational case. While the patient’s prior smoking history and apical bleb formation may suggest smoking-related pulmonary changes, these findings remain associative rather than confirmatory. Nevertheless, this case provides educational value by emphasizing that spontaneous pneumothorax may occur in patients outside the classic demographic profile and highlights the diagnostic complexity that may arise when distinguishing PSP from SSP in former smokers with structural pulmonary abnormalities.

## Conclusions

This case highlights an atypical presentation of spontaneous pneumothorax in an older obese female with a remote smoking history and apical bleb formation. Although causality cannot be established, this case demonstrates the potential diagnostic overlap between PSP and SSP in patients with structural pulmonary abnormalities and prior tobacco exposure. Clinicians should maintain awareness that spontaneous pneumothorax may occur outside the classic demographic profile. Increased recognition of atypical presentations may support earlier diagnosis, informed counseling regarding recurrence risk, and reinforcement of smoking cessation as an important preventive intervention.
